# Low sex hormone binding globulin: a potential predictor of future glucose dysregulation in women

**DOI:** 10.1097/XCE.0000000000000252

**Published:** 2021-07-29

**Authors:** Adrian H. Heald, Ian Laing, Simon Anderson, Mark Livingston

**Affiliations:** a The Faculty of Biology, Medicine and Health and Manchester Academic Health Sciences Centre, University of Manchester; b Department of Diabetes and Endocrinology, Salford Royal Hospital, Salford, UK; c Department of Clinical Biochemistry, Royal Preston Hospital; d University of the West Indies, Cavehill Campus, Barbados; e Department of Clinical Biochemistry, Black Country Pathology Services, Walsall Manor Hospital, Walsall; f School of Medicine and Clinical Practice, University of Wolverhampton, Wolverhampton, UK

**Learning Points**

• Sex hormone binding globulin (SHBG) concentration associates with insulin-resistance.
• We looked at the relation between baseline SHBG concentration and future glycaemic status over an up to 10-year period, for women who were normoglycaemic at baseline.
• In multiple linear regression analysis, a lower SHBG was associated with a higher fasting glucose independent of age and index of multiple deprivation.
• These findings suggest that a low SHBG level, at a relatively young age is a risk marker for future impaired glucose handling in women.

**Precis:**

In a laboratory study, we found that low sex hormone-binding globulin level is a risk marker for future relative dysglycaemia in women, with index of multiple deprivation also an independent predictor of future prediabetes/type 2 diabetes mellitus.

Editor,

Previous work has suggested that a low sex hormone-binding globulin (SHBG) concentration associates with insulin resistance [1]. We previously showed that a low circulating SHBG level (<30 nmol/L) is associated with the presence of the metabolic syndrome in a multiethnic population [2] with lower levels found in both men and women of Pakistan ethnic origin compared with Caucasian and African-Caribbean origin people. In that study SHBG correlated positively with insulin sensitivity as estimated by HOMA-S [3] and negatively with waist-hip ratio, BMI and DBP across all ethnic groups, whereas in multivariate logistic regression analysis a low SHBG increased the likelihood of the metabolic syndrome being present. Furthermore, SHBG levels are often lower in women with polycystic ovarian syndrome (PCOS) [4–6].

There is therefore the potential for SHBG, given its relative ease of measurement (commonly by automated immunoassay), not requiring fasting status or special processing to have utility in identifying people at increased risk or developing impaired glucose handling in the future.

In the light of this, we looked at the predictive value of SHBG in relation to the future development of impaired glucose handling in women in a preliminary study in 115 women, over an up to 10-year period. The women were normoglycaemic at baseline. Outcome was either fasting glucose or glycosylated haemoglobin (HbA1c) [7], as measures of glycaemia.

‘As anonymised data were used for the analysis from a laboratory database and no patient was contacted in this study, approval was sought and obtained from the Research and Innovation team’.

The mean age of the women at the start of follow-up was 26.8 years (SD, 9.0; range, 16–65). Over the up to 10-year follow-up period, 6% developed non-diabetic hyperglycaemia (defined as HbA1c 42–47 mmol/mol) to and 1% type 2 diabetes (T2DM, defined as HbA1c ≥48 mmol/mol). In multiple linear regression analysis, a lower SHBG was associated with higher fasting glucose [normalised beta (β) −0.22; *P* = 0.02] independent of age (β 0.28; *P* = 0.01) and index of multiple deprivation (IMD) (β −0.11; *P* = 0.06) and South Asian ethnicity (β −0.13; *P* = 0.04). In logistic regression, we found that age was predictive of subsequent development of prediabetes/T2DM [odds ratio 1.08 (95% CI, 1.02–1.15); *P* = 0.01] independent of IMD/SHBG/ethnicity.

In the light of these results, we suggest that a low SHBG level may have value as a risk marker for the development of future glucose dysregulation in women, with age also an independent predictor of nondiabetic hyperglycaemia/T2DM. Where someone has been found to have a low SHBG, there is the potential for targeted lifestyle measures to be implemented to prevent or delay the onset of glucose dysregulation.

In our study, a younger age profile of the group likely influenced the low cumulative incidence of T2DM over the 10-year follow-up period. Nevertheless, SHBG may prove to be a useful future marker for the impact of lifestyle change programmes to reduce metabolic risk those people found to have a low SHBG, including women with PCOS.

We plan further work to improve our understanding of this and to continue to follow-up this cohort of women in the coming years.

## Acknowledgements

**Conflicts of interest**

There are no conflicts of interest.
